# Management strategies for CAR-T cell therapy-related toxicities: results from a survey in Greece

**DOI:** 10.3389/fmed.2025.1553966

**Published:** 2025-05-30

**Authors:** Eleni Gavriilaki, Ifigeneia Tzannou, Anna Vardi, Ioannis Tsonis, Maria Liga, Konstantinos Gkirkas, Maria Ximeri, Zoi Bousiou, Maria Bouzani, Eleftheria Sagiadinou, Panagiotis Dolgyras, Nikolaos Kotsiou, Vasiliki Bampali, Despina Mallouri, Tatiana Tzenou, Ioannis Batsis, Damianos Sotiropoulos, Stavros Gigantes, Helen A. Papadaki, Panagiotis Tsirigotis, Alexandros Spyridonidis, Theodoros P. Vassilakopoulos, Maria Angelopoulou, Ioannis Baltadakis, Ioanna Sakellari

**Affiliations:** ^1^Hematology Department-BMT Unit, George Papanikolaou General Hospital, Thessaloniki, Greece; ^2^Department of Hematology-Lymphomas and BMT Unit, Evangelismos Hospital, Athens, Greece; ^3^Department of Internal Medicine - BMT Unit, University Hospital of Patras, Patras, Greece; ^4^BMT Unit, Department of Internal Medicine, Attikon University Hospital, Athens, Greece; ^5^Department of Hematology, University General Hospital of Heraklion, Heraklion, Greece; ^6^German Oncology Center, Limassol, Cyprus; ^7^Department of Hematology-BMT Unit, Laikon General Hospital, Athens, Greece

**Keywords:** CAR-T cells, axicabtagene ciloleucel (axi-cel), tisagenlecleucel (tisa-cel), brexucabtagene autoleucel (brexu-cel), cytokine release syndrome (CRS), immune effector-cell-associated neurotoxicity syndrome (ICANS), dexamethasone, anakinra

## Abstract

Chimeric antigen receptor T-cell (CAR-T) therapy has transformed the management of relapsed or refractory hematologic malignancies, offering remarkable remission rates. However, severe toxicities, including cytokine release syndrome (CRS) and immune effector cell-associated neurotoxicity syndrome (ICANS), are posing challenges to patient care. This multicenter observational study evaluated the prophylactic and treatment strategies for managing severe CRS and ICANS across six transplant centers in Greece. Data from 173 adult patients receiving CAR-T cell products—axi-cel, tisa-cel, and brexu-cel—were analyzed. The incidence of grade 3 CRS was 6.6% for axi-cel, 3.3% for tisa-cel, and 10% for brexu-cel recipients. Grade 4 CRS was documented in 2.5% and 5% in axi-cel and brexu-cel recipients, while grade 5 CRS was recorder only in brexu-cel (10%). Severe ICANS was less frequent, with grade 3 and 4 rates of 7.5% and 2.5% for axi-cel, while brexu-cel documented only grade 3 (10%). Centers utilized prophylactic measures, including levetiracetam and low-dose dexamethasone, significantly reducing severe toxicities. Tocilizumab was administered for CRS management, supplemented by anakinra or siltuximab in select cases. Early intervention strategies effectively minimized progression to severe toxicity. Our findings underscore the importance of standardized prophylactic and therapeutic protocols in mitigating CAR-T-related toxicities. The variability in toxicity incidence reflects differences in patient populations, CAR-T constructs, and clinical practices. Further research is essential to optimize individualized management strategies and advance the safety of CAR-T therapies in clinical settings.

## 1 Introduction

Chimeric antigen receptor T-cell (CAR-T) therapies have revolutionized the treatment for patients with hematologic malignancies, particularly those with relapsed or refractory forms. CAR-T therapy involves the isolation and genetic modification of autologous T-cells in order to express a specific modified T-cell receptor that targets specific antigens on cancer cells, thus enabling a robust immune response (1, 2). First-generation CAR-T cells are characterized by a single-chain variable fragment antigen-recognition domain, a transmembrane domain and a T-cell activation domain derived from CD3. In contrast, second-generation CAR-T cells incorporate an additional co-stimulatory domain, such as CD28 or 4-1BB, while third-generation CAR-T cells feature two co-stimulatory domains, for example, both CD28 and 4-1BB (3). Commercially available second-generation biosynthetic CD19 CAR-T cell products include tisagenlecleucel (tisa-cel) and axicabtagene ciloleucel (axi-cel). Tisa-cel is indicated for the treatment of relapsed or refractory B-ALL in patients up to 25 years of age and for those with relapsed or refractory diffuse large B-cell lymphoma (DLBCL) (4, 5). Conversely, axi-cel is approved for treating refractory DLBCL, Primary Mediastinal Large B-cell Lymphoma (PMBCL) and grade 3B Follicular Lymphoma (6, 7). Additionally, brexucabtagene autoleucel (brexu-cel), another second-generation CD19 CAR-T cell product, has been authorized for the treatment of relapsed or refractory mantle cell lymphoma (MCL) and B-ALL (8, 9).

The approval of these CAR-T products has provided new hope for patients who previously faced limited therapeutic options. Despite their impressive efficacy, which can lead to high rates of complete remission in difficult-to-treat populations, CAR-T therapies are associated with significant complications from the release of cytokines which is triggered by rapid T-cell expansion (10). The most notable toxicities include Cytokine Release Syndrome (CRS) and Immune effector Cell-Associated Neurotoxicity Syndrome (ICANS). CRS is characterized by a systemic inflammatory response due to the rapid activation and proliferation of CAR-T cells, leading to symptoms ranging from mild fever to severe complications such as hypotension and multi-organ failure (3, 10). Similarly, ICANS presents neurological symptoms that can range from confusion and delirium to seizures and coma (10, 11). These toxicities pose substantial challenges in clinical management and can significantly impact patient outcomes. CRS is mostly observed within the first week of infusion and affects a variable percentage of patients (30–90%), while ICANS affects around 40–65% of individuals (12). It is noteworthy that CAR-T products incorporating a CD28 co-stimulatory domain are primarily linked with ICANS, while those utilizing a 4-1BB domain tend to be more associated with CRS (3, 13).

The pathophysiology underlying CRS and ICANS is complex. Emerging evidence suggests that these syndromes may share similarities with endothelial injury syndromes observed in other contexts, such as post-hematopoietic cell transplantation (HCT). The interplay between inflammatory cytokines, endothelial dysfunction, and hypercoagulability appears to be a common theme across these conditions (14–16). As a result, markers of endothelial activation (Endothelial Activation and Stress Index – EASIX and its modified version m-EASIX) have been proposed as predictors of ICANS, CRS, and overall survival (OS) in patients receiving CAR-T cell therapy (17).

Current management strategies primarily involve corticosteroids and interleukin-6 (IL-6) inhibitors like tocilizumab (18–21). However, there is considerable variability in practice across different centers regarding prophylactic measures and treatment protocols. Recent literature has explored various strategies aimed at mitigating the risks associated with these toxicities. For instance, studies have indicated that early intervention with tocilizumab and/or steroids with lower grade toxicity, can significantly prevent onset of more severe CRS and its subsequent effects on neurotoxicity (22, 23). Additionally, prophylactic treatment regardless of toxicity observation has been proposed (24). Furthermore, the use of anakinra, a recombinant IL-1 receptor antagonist, and siltuximab, another chimeric anti-IL-6 monoclonal antibody, has been also investigated as a potential treatment for severe CRS and ICANS, showing promise in reducing inflammatory responses without the adverse effects associated with steroids (25–27).

The objective of this study is to document the prophylactic and treatment strategies employed for managing severe CRS and ICANS in real-world clinical settings across six transplant centers. By analyzing data from these centers, we seek to provide insights into current practices, highlight areas for improvement, and ultimately contribute to the optimization of patient care in this rapidly evolving field.

## 2 Methods

This multi-center observational study was conducted through an online survey distributed across six transplant centers which administer CAR-T therapies in Greece. The primary objective of the survey was to gather comprehensive data on administration of specific CAR-T products, incidence and grading of CRS and ICANS, implementation of treatment protocols and prophylactic measures, and patient outcomes. Data were collected from consecutive adult patients (≥18 years) diagnosed with relapsed or refractory lymphomas or B-ALL who received commercially available CAR-T cell products in accordance with established clinical guidelines. Ethnicity data were collected at the time of CAR T-cell therapy administration. All patients in the cohort self-identified as White of European heritage. Data were reported until the end of June 2024, with a minimum follow-up of 1-month post-infusion. Results are presented with the use of descriptive statistics with IBM SPSS Statistics 22.0.

All individuals received lymphodepleting therapy prior to CAR-T cell infusion, with cyclophosphamide and fludarabine, in alignment with each product’s specific protocol (28). Tisa-cel and axi-cel have been administered since 2020, while brexu-cel was introduced in 2022. Patient monitoring was conducted in collaboration with neurologists and intensive care specialists, adhering to the guidelines established by the European Society for Blood and Marrow Transplantation (EBMT) and MD Anderson Cancer Center (28, 29). Diagnosis and grading of CRS and ICANS were performed according to the American Society for Transplantation and Cellular Therapy (ASTCT) grading system, with grades ≥ 3 classified as severe (30). Prophylactic treatment with levetiracetam was administered to all CAR-T cell recipients starting on the day of infusion (31). The studies involving humans were approved by Institutional Review Board of all six transplant centers (George Papanikolaou General Hospital, Evangelismos Hospital, University Hospital of Patras, Attikon University Hospital, General Hospital of Heraklion, Laikon General Hospital). The studies were conducted in accordance with the local legislation and institutional requirements (32). Written informed consent for participation was not required from the participants or the participants’ legal guardians/next of kin in accordance with the national legislation and institutional requirements.

## 3 Results

A total of 173 adult patients received commercially available CAR-T cell products, with 120 patients treated with axi-cel (69.4%), 33 patients receiving tisa-cel (19.1%), and 20 patients brexu-cel (11.5%). The median age at CAR T-cell infusion was 50 years (range, 18–77). All 173 patients included in the study were of White European descent. Most patients (156/173; 90.2%) were diagnosed with non-Hodgkin lymphoma (NHL), while 17 patients (9.8%) had B-cell acute lymphoblastic leukemia (B-ALL). Among the NHL subtypes, diffuse large B-cell lymphoma (DLBCL) was the most common diagnosis, observed in 105 patients (60.7%), followed by primary mediastinal B-cell lymphoma (PMBCL) in 26 patients (15.1%), transformed follicular lymphoma (TFL) in 16 patients (9.2%), and mantle cell lymphoma (MCL) in 9 patients (5.2%). Regarding prior treatment history, patients had received a median of 3 prior lines of therapy (range: 1–9), with 28 individuals (16.2%) having undergone autologous hematopoietic stem cell transplantation and 15 patients (8.7%) having received allogeneic transplantation (Table 1).

**TABLE 1:** Overview of patient characteristics and CAR-T products.

Patient characteristics	Total (*N* = 173)
Age at CAR T-cell infusion, median (range), y	50 (18–77)
**Ethnicity**	
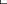 White, European descent	173 (100%)
**Diagnosis, *n* (%)**	
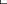 B-ALL	17 (9.8%)
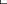 NHL	156 (90.2%)
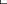 DLBCL	105 (60.7%)
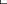 PMBCL	26 (15.1%)
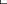 TFL	16 (9.2%)
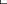 MCL	9 (5.2%)
**CAR T-cell product, *n* (%)**	
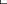 Axicabtagene ciloleucel	120 (69.4%)
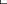 CRS grade 3	8 (6.6%)
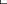 CRS grade 4	3 (2.5%)
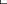 ICANS grade 3	9 (7.5%)
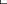 ICANS grade 4	3 (2.5%)
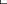 Tisagenlecleucel	33 (19.1%)
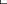 CRS grade 3	1 (3.3%)
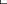 Brexucabtagene autoleucel	20 (11.5%)
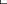 CRS grade 3	2 (10%)
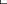 CRS grade 4	1 (5%)
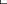 CRS grade 5	2 (10%)
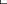 ICANS grade 3	2 (10%)
Prior lines of treatment, median (range)	3 (1–9)
Previous autologous transplantation	28 (16.2%)
Previous allogeneic transplantation	15 (8.7%)

B-ALL, B-cell acute lymphoblastic leukemia; DLBCL, diffuse large B-cell lymphoma; MCL, mantle cell lymphoma; NHL, non-Hodgkin lymphoma; PMBCL, primary mediastinal large B-cell lymphoma; TFL, transformed follicular lymphoma.

**FIGURE 1:**
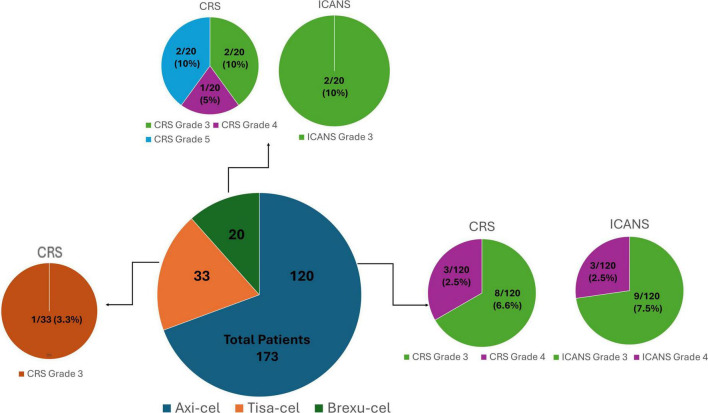
Incidence and severity of toxicities after CAR-T cell therapy.

Toxicity outcomes varied across products. The incidence of Grade 3 CRS was observed in 8 out of 120 (6.6%) axi-cel recipients, in 1 out of 33 (3.3%) tisa-cel recipients and in 2 out of 20 (10%) brexu-cel recipients. Grade 4 CRS was recorded in 3 out of 120 (2.5%) patients treated with axi-cel and in 1 out of 20 (5%) brexu-cel recipients. Notably, Grade 5 CRS was observed exclusively among those receiving brexu-cel, affecting 2 out of 20 individuals (10%) (Figure 1). 2 centers implemented low-dose dexamethasone as a prophylactic treatment, administering a total dose of 30 mg to 18 patients receiving axi-cel and a total dose of 40 mg to an additional 13 axi-cel recipients; among these individuals, only 3 developed severe toxicity, each experiencing Grade 3 CRS. All centers utilized tocilizumab at a dosage of 8 mg/kg for a median of 4 doses (range: 3–6), even for cases classified as Grade 1 CRS lasting longer than 24 h. If symptoms persisted without improvement, dexamethasone was given at a dosage of 10 mg per dose, with a median total dose of 40 mg (range: 10–150). Additionally, anakinra was administered by 2 centers at a dosage of 100 mg every 6 h, while siltuximab was used by 1 single center (Table 2).

**TABLE 2:** Treatment strategies and administration details.

Intervention	Details
Prophylactic dexamethasone	Administered in 2 centers to 31 axi-cel recipients
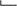 30 mg total dose	18 patients
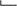 40 mg total dose	13 patients
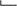 Severe CRS among prophylaxis group	3 patients (Grade 3 CRS only)
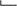 Severe ICANS among prophylaxis group	3 patients (no concurrent severe CRS)
Prophylactic levetiracetam	Administered to all CAR-T recipients for seizure prevention
Tocilizumab	Used in all centers for CRS
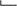 Dose	8 mg/kg
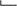 Median number of doses	4 (range: 3–6)
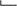 Indication	Administered for CRS Grade ≥ 1 lasting > 24 h
Therapeutic dexamethasone (CRS)	Used when CRS symptoms persisted after tocilizumab
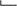 Dose per administration	10 mg
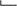 Median total dose	40 mg (range: 10–150)
Therapeutic dexamethasone (ICANS)	All patients with severe ICANS received dexamethasone
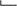 Median total dose	30 mg (range: 10–240)
Methylprednisolone (ICANS)	Used in 2 patients with prior prophylactic dexamethasone
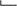 Median total dose	2 gr (range: 1–4 gr)
Anakinra	Administered in 2 centers
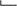 Dose	100 mg every 6 h
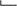 Used for	Refractory CRS or ICANS
Siltuximab	Used in 1 center
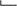 Used in	1 patient with concurrent severe CRS and ICANS

Regarding neurotoxicity, ICANS grade 3 was reported in 9 out of 120 (7.5%) axi-cel recipients and in 2 out of 20 (10%) brexu-cel recipients. Grade 4 ICANS was documented in 3 out of the 120 patients treated with axi-cel (2.5%) (Table 1). Among those experiencing severe ICANS, 3 had received prophylactic dexamethasone and did not exhibit severe CRS (Grade ≥ 3). All patients with severe ICANS were treated with dexamethasone, receiving a median total dose of 30 mg (range: 10–240). Methylprednisolone was administered at a median total dose of 2 gr (range: 1–4) in 2 patients who had previously received low-dose dexamethasone for CRS. At a single center, 1 patient presenting both severe CRS and ICANS received anakinra and siltuximab (Table 2). Regarding the prognostic markers, 2 centers employed the Endothelial Activation and Stress Index (EASIX), along with bedside electroencephalogram (EEG) monitoring in 1 center. Lastly, another center utilized management algorithms developed by the French group DESCAR-T to inform treatment decisions (33).

## 4 Discussion

The findings of our study provide critical insights into the incidence and management of complications among patients receiving CAR-T cell therapy. We evaluated 173 adult patients across 6 transplant centers, documenting the incidence of CRS and ICANS following the administration of commercial CAR-T products, specifically tisa-cel, axi-cel and brexu-cel. Our results indicated a low incidence of CRS and ICANS occurrence. These findings underscore the importance of effective management strategies, including the use of prophylactic measures and early intervention with steroids and interleukin inhibitors like siltuximab and anakinra.

In various clinical trials, the incidence of all grade CRS has been reported between 30 and 90%, while ICANS affects approximately 40%–65% of patients receiving CAR-T cell therapy (12, 34). The lower rates observed in our cohort may reflect a combination of factors including patient selection, the specific CAR-T products administered and the implementation of prophylactic measures. Notably, while our study reported a lower incidence of severe CRS compared to some trials (35, 36), it aligns with other studies that have documented comparable outcomes for specific CAR-T constructs (26, 37, 38). The variability in incidence rates across studies may be attributed to differences in patient demographics, conditioning regimens, and institutional protocols for monitoring and managing these toxicities (39). However, due to the survey nature of our study and the absence of granular, patient-level data linking each specific demographic or disease feature directly to toxicity outcomes, definitive conclusions regarding the impact of these variables on toxicity cannot be drawn in our cohort. Future studies from our team with more detailed individual patient data are warranted to better delineate these relationships. The administration of brexu-cel was limited to a smaller patient population, which also constrains our ability to draw definitive conclusions regarding its safety profile.

The management strategies observed in our study reflect a real-world, multi-center approach to mitigate CAR-T cell therapy-related toxicities. Prophylactic measures varied among centers, with all institutions uniformly administering levetiracetam for seizure prophylaxis in the context of ICANS. Importantly, low-dose dexamethasone was used prophylactically in a subset of patients at 2 centers, involving 31 individuals receiving axi-cel. Among this subgroup, only 3 patients developed severe CRS (all Grade 3), suggesting a potential protective effect. This observation supports emerging evidence that early corticosteroid use may help reduce the incidence or severity of CAR-T-related toxicity without compromising efficacy (24, 40–42).

Tocilizumab use was consistent across all participating centers and was administered in response to persistent Grade 1 CRS lasting more than 24 h, reflecting a proactive, early-intervention approach. This aligns with current guidelines and literature stating its established role in alleviating symptoms associated with cytokine release (43–45). In more complex or refractory cases, additional interleukin inhibitors (anakinra/siltuximab) were used. Anakinra was administered at 100 mg every 6 h in 2 centers, while siltuximab was used in 1 center. Although these agents were used in a limited number of cases, outcomes suggested a beneficial proactive approach particularly towards controlling severe ICANS or overlapping CRS/ICANS when corticosteroids and tocilizumab were insufficient, a strategy that has been also implemented in other studies (26, 46, 47). This points to the potential value of incorporating IL-1 and IL-6 blockade into treatment algorithms for high-risk patients.

To support clinical decision-making, several centers also implemented diagnostic and monitoring tools. Specifically, 2 centers employed EASIX as a marker for risk stratification, while 1 center utilized bedside EEG monitoring. In addition, a management algorithm developed by the French DESCAR-T group was used in 1 institution, facilitating structured and timely treatment decisions (33). These practices reflect the increasing importance of data-driven, algorithm-based approaches to early intervention and toxicity management (18).

Taken together, our data support the concept of an integrated management approach to CAR-T cell toxicities, one that combines universal prophylaxis (e.g., levetiracetam), early pharmacologic intervention (e.g., tocilizumab, corticosteroids), and selective use of advanced immunomodulatory agents. These strategies, complemented by continuous monitoring and risk-based stratification, are essential for improving patient safety and outcomes (48).

While these findings offer valuable insights, they should be interpreted in the context of the study’s limitations, including the retrospective survey design and the lack of patient-level data, which prevent statistical correlation between interventions and outcomes. Looking ahead, larger prospective studies are needed to validate these real-world findings and to better define optimal combinations and timing of prophylactic and therapeutic interventions. Nonetheless, our study provides a meaningful foundation for supporting the adoption of proactive, individualized, and standardized strategies in CAR-T therapy management.

## 5 Conclusion

Our study contributes valuable data on the incidence rates of CRS and ICANS among patients receiving CAR-T cell therapy while highlighting effective management strategies involving steroids and interleukin inhibitors. The variability in reported incidence rates across different studies emphasizes the necessity for standardized monitoring protocols to enhance patient safety. Furthermore, our findings advocate for proactive prophylactic measures as essential components of care in mitigating the risks associated with CAR-T cell therapy toxicities. As the landscape evolves with emerging treatments like bispecific antibodies that may require different prophylactic measures, it is critical that we continue to refine our understanding for these life-saving therapies. Future research should continue to explore optimal management strategies tailored to individual patient needs while considering advancements in CAR-T technology that may influence toxicity profiles. Continued collaboration among transplant centers will be crucial in refining treatment protocols and improving outcomes for patients undergoing CAR-T cell therapy.

## 6 Limitations

The survey-based design of our study did not allow for the collection of patient-level data, preventing statistical analyses of specific demographic or clinical factors in relation to toxicity outcomes. Additionally, the cohort was ethnically homogeneous, with all patients being of White European descent, which may limit the generalizability of our findings to more diverse populations. The number of patients who received brexu-cel was small (*n* = 20), restricting our ability to draw firm conclusions about its safety profile. The retrospective, self-reported nature of the data may also introduce reporting bias or inconsistencies in toxicity grading. Moreover, important clinical variables such as performance status, comorbidities, and details on bridging therapy were not captured, further limiting the depth of our analysis. Despite these limitations, our findings offer meaningful real-world insights and highlight areas for further investigation in prospective, standardized studies.

## Data Availability

The original contributions presented in this study are included in this article/supplementary material, further inquiries can be directed to the corresponding author.
